# Chiral quantum heating and cooling with an optically controlled ion

**DOI:** 10.1038/s41377-024-01483-5

**Published:** 2024-06-26

**Authors:** Jin-Tao Bu, Jian-Qi Zhang, Ge-Yi Ding, Jia-Chong Li, Jia-Wei Zhang, Bin Wang, Wen-Qiang Ding, Wen-Fei Yuan, Liang Chen, Qi Zhong, Ali Keçebaş, Şahin K. Özdemir, Fei Zhou, Hui Jing, Mang Feng

**Affiliations:** 1 State Key Laboratory of Magnetic Resonance and Atomic and Molecular Physics, Wuhan Institute of Physics and Mathematics, Innovation Academy of Precision Measurement Science and Technology, Chinese Academy of Sciences, 430071 Wuhan, China; 2 University of the Chinese Academy of Sciences, 100049 Beijing, China; 3 Research Center for Quantum Precision Measurement, Guangzhou Institute of Industry Technology, 511458 Guangzhou, China; 4 Department of Engineering Science and Mechanics, and Materials Research Institute, Pennsylvania State University, University Park, State College, PA 16802 USA; 5 Key Laboratory of Low-Dimensional Quantum Structures and Quantum Control of Ministry of Education, Department of Physics and Synergetic Innovation Center for Quantum Effects and Applications, Hunan Normal University, 410081 Changsha, China; 6 Department of Physics, Zhejiang Normal University, 321004 Jinhua, China

**Keywords:** Quantum optics, Atom optics

## Abstract

Quantum heat engines and refrigerators are open quantum systems, whose dynamics can be well understood using a non-Hermitian formalism. A prominent feature of non-Hermiticity is the existence of exceptional points (EPs), which has no counterpart in closed quantum systems. It has been shown in classical systems that dynamical encirclement in the vicinity of an EP, whether the loop includes the EP or not, could lead to chiral mode conversion. Here, we show that this is valid also for quantum systems when dynamical encircling is performed in the vicinity of their Liouvillian EPs (LEPs), which include the effects of quantum jumps and associated noise—an important quantum feature not present in previous works. We demonstrate, using a Paul-trapped ultracold ion, the first chiral quantum heating and refrigeration by dynamically encircling a closed loop in the vicinity of an LEP. We witness the cycling direction to be associated with the chirality and heat release (absorption) of the quantum heat engine (quantum refrigerator). Our experiments have revealed that not only the adiabaticity breakdown but also the Landau–Zener–Stückelberg process play an essential role during dynamic encircling, resulting in chiral thermodynamic cycles. Our observations contribute to further understanding of chiral and topological features in non-Hermitian systems and pave a way to exploring the relation between chirality and quantum thermodynamics.

## Introduction

Quantum heat engines (QHEs), using quantum matter as their working substance, convert the heat energy from thermal reservoirs into useful work. QHEs have been implemented in various microscopic and nanoscopic systems, including single trapped ions and spin ensembles^1–7^. Routes to realize QHEs in superconducting circuits^8^ and quantum optomechanical systems^9,10^ have also been proposed. Quantum refrigerators (QRs) are typically achieved by reversing the sequence of the strokes of QHEs^11,12^, removing heat from the cold bath at the expense of external work performed on the system. QRs have been realized with superconducting qubits^13,14^ quantum dots^15^ and trapped ions^16,17^.

Another emergent field attracting widespread interest is non-Hermitian dynamics in classical and quantum systems and exotic features associated with non-Hermitian spectral degeneracies known as exceptional points (EPs)^18–26^. In contrast to Hermitian spectral degeneracies, where eigenvectors associated with degenerate eigenvalues are orthogonal, at an EP, both the eigenvalues and the associated eigenvectors become degenerate. The location of the EPs in the parameter space of a system is typically calculated from the system’s Hamiltonian, which describes the non-unitary coherent evolution of the open system. Such EPs are thus referred to as Hamiltonian EPs (or HEPs). However, HEPs do not take quantum jumps and the associated noise into account and, therefore, do not depict the whole dynamics of an open quantum system. Instead, one should resort to Liouvillian formalism to describe both the non-unitary evolution and the decoherence and, hence, the quantum jumps. Consequently, the EPs of the system are defined as the eigenvalue degeneracies of Liouvillian superoperators—and thus Liouvillian EPs (or LEPs)^27–31^.

Open quantum systems exchange energy with external thermal baths, leading to quantum jumps. In the presence of quantum jumps, the dynamics of QHEs and QRs can be fully described and well understood using a non-Hermitian framework based on Liouvillian formalism and LEPs, especially for the QHEs based on qubits. Moreover, LEPs introduce unique physical properties to QHEs, such as the presence of an LEP can optimize the dynamics of QHEs towards their steady states^30^, enhance the QHE efficiency^32^, and endow topological properties to the QHE^33^. Nevertheless, LEPs and effects associated with the presence of LEPs have remained largely unexplored in quantum systems and, in particular, in the field of quantum thermodynamics.

Dynamically encircling an HEP in a parametric loop has been shown to give rise to chiral state transfer due to non-Hermiticity induced non-adiabatic transitions^34–41^. However, recent experimental studies have shown that chiral behavior can be observed even without encircling a HEP: Any dynamically formed parametric loop in the vicinity of an EP should result in chiral features thanks to the eigenvalue landscape close to the EP^42–46^. Meanwhile, the eigenvalue landscape of an LEP exhibits similar Riemann surfaces, leading to non-trivial state transfer dynamics (e.g., entangled state generation) when the LEP is encircled^31,47^. Some experimental studies illustrate the challenge of exploring the counterintuitive chiral behavior in quantum systems without encircling the LEPs^33,48–50^. For example, the topology and landscape of the Riemann surface, along with the trajectory and evolution speed of the dynamical process, significantly influence the results of parametric loops^33^. These influences result from various aspects, including the phases of the Landau–Zener–Stückelberg (LZS) process^48–50^, quantum coherence, network, and efficiency of the quantum heat engine.

In this Letter, we experimentally demonstrate chiral behavior in a qubit system without encircling LEPs. Namely, we show chiral operation induced by parametric loops in the vicinity of an LEP (without encircling it) in the parameter space of a single trapped ion configured as a quantum engine for heating and refrigeration. Our work brings together quantum thermodynamics, LEPs, and chiral state transfer due to the breakdown of adiabaticity, demonstrating that non-adiabaticity and LZS process^48–50^ are essential to chiral thermodynamic cycles. Our experiment connects, for the first time, the LZS process to chirality in association with LEP-related thermodynamic effects.

## Results

### Experimental setup

**Fig. 1:**
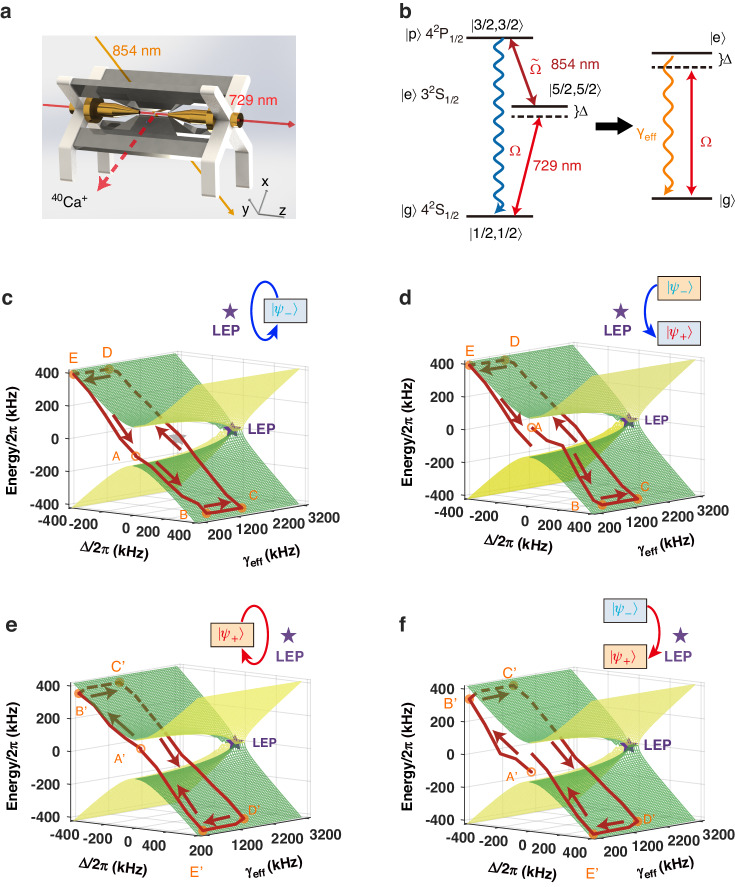
Chiral dynamics for parametric loops in the vicinity of an LEP in the parameter space of a single trapped ion. **a** The linear Paul trap. **b** Level scheme of the ion, where the solid arrows represent the transitions with Rabi frequencies $\Omega$ and $\widetilde{\Omega }$ driven by 729 and 854 nm lasers, respectively, and $\Delta$ is the detuning between the energy level and 729 nm laser. The wavy arrow denotes the spontaneous emission with decay rate $\Gamma$. This three-level model can be simplified to an effective two-level system with tunable drive and decay. **c**–**f** Trajectories without encircling the LEP on the Riemann sheets when completing a clockwise or counterclockwise encirclement in $\triangle -{\gamma }_{{\rm{eff}}}$ parametric space starting from $\left|{\psi }_{+}\right\rangle$ or $\left|{\psi }_{-}\right\rangle$, where the parameters take the values ${\Delta }_{\min }/2\pi =$ 400 kHz, ${{\rm{\gamma }}}_{\min }\approx 100$ kHz, ${{\rm{\gamma }}}_{\min }\approx 1.45$ MHz. The empty circles represent the starting points. Five orange corner points A–E (A’–E’) are labeled for convenience of description in the text. The LEPs are labeled by stars

Our experiment is carried out in a single ultracold ^40^Ca^+^ ion confined in a linear Paul trap (Fig. 1a with more details in refs. ^51,52^) whose axial and radial frequencies are, respectively, ${\omega }_{z}/2\pi =1.01$ and ${\omega }_{r}/2\pi =1.2$ MHz under the pseudo-potential approximation. Under an external magnetic field of 0.6 mT directed in axial orientation, the ground state $\left|{4}^{2}{S}_{1/2}\right\rangle$ is split into two hyperfine energy levels while the metastable state $\left|{3}^{2}{D}_{5/2}\right\rangle$ is split into six. As shown in Fig. 1b, we label $\left|{4}^{2}{S}_{1/2},{m}_{J}=+3/2\right\rangle$ as $\left|g\right\rangle$, $\left|{3}^{2}{D}_{5/2},{m}_{J}=+5/2\right\rangle$ as $\left|e\right\rangle$ and $\left|{4}^{2}{P}_{3/2},{m}_{J}=+3/2\right\rangle$ as $\left|p\right\rangle$. After Doppler and resolved sideband cooling of the ion, we reduce the average phonon number of the *z*-axis motional mode of the ion to be much smaller than 1 with the Lamb–Dicke parameter ∼0.11, which is sufficient to avoid detrimental effects of thermal phonons (e.g., Rabi oscillation offsets). Introducing the dipolar transition $\left|e\right\rangle$ → $\left|p\right\rangle$ by switching on 854-nm laser as explained in refs. ^32,53^, we reduce this three-level system to an effective two-level system representing a qubit. (In this model, we can engineer both the Rabi frequency Ω and the effective decay rate *γ*_eff_ = $\widetilde{\Omega }$ 2/Γ under the condition of Ω ≪ $\widetilde{\Omega }$^32,53^. With this level of controllability, we can fully tune this two-level system and perform parametric loops that encircle or do not encircle the LEP). We employ this qubit as the working substance of the QHE and QR.

The dynamical evolution of this effective two-level model is governed by the Lindblad master equation, $\dot{\rho}={\mathcal{L}}\rho =-i\left[{H}_{{\rm{eff}}},\rho \right]+\frac{{\gamma }_{{\rm{eff}}}}{2}(2{\sigma }_{-}\rho {\sigma }_{+}-{\sigma }_{+}{\sigma }_{-}\rho -{\rho \sigma }_{+}{\sigma }_{-})$, where ${\mathcal{L}}$ is a Liouvillian superoperator, and $\rho$ and ${\gamma }_{{\rm{eff}}}$ denote the density operator and the effective decay rate from |e〉 to |g〉, respectively. Here, the effective Hamiltonian is ${H}_{{\rm{eff}}}=\Delta \left|e\right\rangle \left\langle e\right|+\frac{\Omega }{2}(\left|e\right\rangle \left\langle g\right|+\left|g\right\rangle \left\langle e\right|)$, where $\Delta$ represents the frequency detuning between the resonance transition and the driving laser while $\Omega$ denotes the Rabi frequency. The eigenvalues of ${\mathcal{L}}$ at $\Delta =0$ are given by ${\lambda }_{1}=0$, ${\lambda }_{2}={-\gamma }_{{\rm{eff}}}/2$, ${\lambda }_{3}={(-3\gamma }_{{\rm{eff}}}-\xi )/4$ and ${\lambda }_{4}={(-3\gamma }_{{\rm{eff}}}+\xi )/4$, with $\xi =\sqrt{{\gamma }_{{\rm{eff}}}^{2}-16{\Omega }^{2}}$. It is evident that the eigenvalues ${\lambda }_{3}$ and ${\lambda }_{4}$ coalesce when ${\gamma }_{{\rm{eff}}}=4\Omega$, giving rise to a second order LEP at $\widetilde{\lambda }={-3\gamma }_{{\rm{eff}}}/4$ (see Supplementary Materials). In the weak coupling situation ${\gamma }_{{\rm{eff}}}\, > \,4\Omega$, both ${\lambda }_{3}$ and ${\lambda }_{4}$ are real with a splitting of $\xi /2$. This regime corresponds to the broken phase characterized by a non-oscillatory dynamics accompanied by purely exponential decay^28,29^. In the strong coupling regime (${\gamma }_{{\rm{eff}}} \,< \,4\Omega$), ${\lambda }_{3}$ and ${\lambda }_{4}$ are a pair of complex conjugates with the imaginary parts—$\xi /4$ and $\xi /4$, respectively. This regime corresponds to the exact phase characterized by an oscillatory dynamics. Thus, LEP acts as a critical damping point of the damped harmonic oscillator, which divides the parameter space into a region of oscillatory dynamics (exact phase, ${\gamma }_{{\rm{eff}}}\, < \,4\Omega$) and a region of non-oscillatory dynamics (broken phase, ${\gamma }_{{\rm{eff}}}\, > \,4\Omega$).

Our QHE and QR cycles differ from their classical counterparts in the definition and implementation of the characteristic thermodynamic quantities. The working substance in our QHE and QR is the qubit defined above. The thermal baths consist of the 729-nm laser irradiation and the actual environment. This working substance carries out work by varying the detuning $\Delta$ in the effective Hamiltonian ${H}_{{\rm{eff}}}$, in which the increase and decrease of the population in the excited state |e〉 correspond to heat absorption and heat release of the working substance, respectively. As a result, the Rabi interactions in the QHE and QR cycles represent the energy exchange between the working substance and thermal baths. Variations in the effective Hamiltonian lead to LZS process, along with performed work, heat absorption, and heat release^33^. In our experiments, all thermodynamic quantities are acquired from population variations of the qubit and the corresponding tunable parameters (see Supplementary Materials).

Figure 1a, b presents the thermodynamic cycle we carry out, consisting of two iso-decay and two isochoric strokes with a fixed Rabi frequency $\Omega$ as in ref. ^33^ For the iso-decay strokes, we achieve positive (negative) work by decreasing (increasing) the detuning $\Delta$ while keeping the decay rate ${\gamma }_{{\rm{eff}}}$ constant. The iso-decay strokes shift the energy difference between the two levels of the working substance and function as the expansion and compression processes for the work done. In the isochoric strokes, our system rapidly reaches a steady state. Then, with the constant values of $\triangle$ and $\Omega$, we increase (or decrease) the population of the excited state for heating (or cooling) by decreasing (increasing) the decay rate ${\gamma }_{{\rm{eff}}}$. This indicates that the increase (decrease) of the population in the excited state corresponds to the heat absorption (release) from (to) the thermal baths.

To study the effect of the parametric loops in the vicinity of the LEP on the thermodynamic cycle and the engine performance, we prepare the qubit in the superposition state $\left|{\psi }_{+}\right\rangle =(\left|e\right\rangle +\left|g\right\rangle )/\sqrt{2}$ or $\left|{\psi }_{-}\right\rangle =(\left|e\right\rangle -\left|g\right\rangle )/\sqrt{2}$, and then perform a thermodynamic cycle, corresponding to a loop in the parameter space by tuning ${\gamma }_{{\rm{eff}}}$ and $\Delta$ such that the LEP of the system is not encircled. The superposition states $\left|{\psi }_{+}\right\rangle$ and $\left|{\psi }_{-}\right\rangle$ correspond to two eigenstates of ${H}_{{\rm{eff}}}$ for ∆ = 0 kHz at the middle point of the expansion and compression processes in the iso-decay strokes, respectively. Moreover, to prevent our operations from affecting the vibrational modes of the ion, leading to unexpected heating noises, we restrict $\left|\Delta \right|$ to be smaller than half of the vibrational frequency.

Starting from the mid-points of these strokes requires five strokes to execute a clockwise (CW) or a counterclockwise (CCW) loop. After the loop (CW or CCW) is completed, we compare the final state $\rho$ of the qubit with the initial state $\left|{\psi }_{+}\right\rangle$ or $\left|{\psi }_{-}\right\rangle$ by calculating the fidelity ${\rm{\langle }}{\psi }_{+}{\rm{|}}\rho \left|{\psi }_{+}\right\rangle$ or ${\rm{\langle }}{\psi }_{-}{\rm{|}}\rho \left|{\psi }_{-}\right\rangle$. Figure 1c, d show the evolution of the system for two counterclockwise loops that start at the initial states $\left|{\psi }_{-}\right\rangle$ and $\left|{\psi }_{+}\right\rangle$, respectively, and are completed without encircling the LEP. We first implement an iso-decay compression stroke from A to B by increasing the detuning $\Delta$ linearly from 0 to its maximum value ${\Delta }_{\max }$ while keeping ${\gamma }_{{\rm{eff}}}$ at its minimum value of ${\gamma }_{\min }$. The second stroke is an isochoric cooling stroke from B $({\Delta}_{\max},{\gamma}_{\min})$ to C $({\Delta }_{\max },{\gamma }_{\max })$, which is realized by increasing ${\gamma }_{{\rm{eff}}}$ from ${\gamma }_{\min }$ to ${\gamma }_{\max }$ with the detuning remaining constant at ${\Delta }_{\max }$. Then we execute the third stroke, i.e., an iso-decay expansion from C $({\Delta }_{\max },{\gamma }_{\max })$ to D $({\Delta }_{\min },{\gamma }_{\max })$, by decreasing $\Delta$ from ${\Delta }_{\max }$ to ${\Delta }_{\min }$ while keeping ${\gamma }_{{\rm{eff}}}={\gamma }_{\max }$. The fourth stroke is an isochoric heating from D $({\Delta }_{\min },{\gamma }_{\max })$ to E $({\Delta }_{\min },{\gamma }_{\min })$ executed by decreasing ${\gamma }_{{\rm{eff}}}$ from ${\gamma }_{\max }$ to ${\gamma }_{\min }$ with $\Delta$ fixed at ${\Delta }_{\min }$. The final step is an iso-decay compression stroke from E $({\Delta }_{\min },{\gamma }_{\min })$ back to A$(0,{\gamma }_{\min })$ executed by increasing $\Delta$ from ${\gamma }_{{\rm{eff}}}$ to 0 while ${\gamma }_{{\rm{eff}}}$ is kept fixed at ${\gamma }_{\min }$. The clockwise loops depicted in Fig. 1e, f are also executed in five strokes with the initial states prepared in $\left|{\psi }_{+}\right\rangle$ and $\left|{\psi }_{-}\right\rangle$ but in reverse order of the process described above for counterclockwise loops. It is clearly seen that regardless of the initial state that the loops start from, the system always ends up at the final state $\left|{\psi }_{+}\right\rangle$ for a clockwise loop and at the final state $\left|{\psi }_{-}\right\rangle$ for a counterclockwise loop. This observation that the execution direction of the loop determines the final state is a signature of the chiral behavior in our system. This process is often referred to as asymmetric mode conversion or chiral state transfer^36,46,48^.

### Quantum heating and cooling

**Fig. 2:**
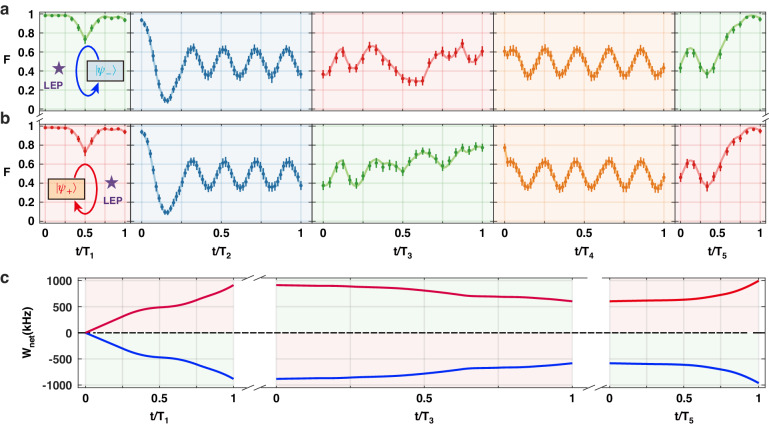
Closed loops that lead to QHE and QR. **a** Counterclockwise loop for the QR starting from $\left|{\psi }_{-}\right\rangle$. **b** Clockwise loop for the QHE starting from $\left|{\psi }_{+}\right\rangle$. Evolution of the system’s state along the trajectory of the closed loop is characterized by the fidelity $\langle {\psi }_{-}|\rho (t)\left|{\psi }_{-}\right\rangle$ in (**a**) and the fidelity $\langle {\psi }_{+}|\rho (t)\left|{\psi }_{+}\right\rangle$ in (**b**). The circles and error bars denote, respectively, the average and standard deviations of 10,000 measurements. The solid curves connecting the dots are obtained by simulating master equations. **c** Blue and red solid curves represent, respectively, the time evolution of the network in the counterclockwise and clockwise loops, where only three of the strokes associated with doing work are depicted. Durations of the five strokes are *T*_1_ = *T*_5_ = 6 μs, *T*_3_ = 12 μs, and *T*_2_ = *T*_4_ = 150 μs. Other parameters are Ω/2*π* = 120 kHz, Δ_min_/2*π* = −400 kHz, Δ_max_/2*π* = 400 kHz, *γ*_min_ ≈ 0 kHz, and *γ*_max_ ≈ 1.45 MHz

Figure 2 displays experimental results of a counterclockwise loop starting at $\left|{\psi }_{-}\right\rangle$ and a clockwise loop starting at $\left|{\psi }_{+}\right\rangle$, corresponding to the schemes in Fig. 1c, e. These loops do not encircle the LEP of the system. In Fig. 2b, we prepare the qubit in the initial state $\left|{\psi }_{+}\right\rangle$ with fidelity $\langle {\psi }_{+}|{\rho }_{A}\left|{\psi }_{+}\right\rangle$ = 0.985 which is lower than 1 due to imperfections during the state preparation. The system returns to $\left|{\psi }_{+}\right\rangle$ after completion of the loop. Figure 2a exhibits the results of the counterclockwise loop starting at $\left|{\psi }_{-}\right\rangle$. The state of the system evolves back to the initial state $\left|{\psi }_{-}\right\rangle$ when the loop is completed. The two situations correspond, respectively, to the QR and QHE cycles, as elucidated later. For the counterclockwise encirclement in Fig. 2a, the system is initialized to $\left|{\psi }_{-}\right\rangle =(\left|e\right\rangle -\left|g\right\rangle )/\sqrt{2}$. The Landau-Zener transition occurs in the first stroke with the detuning $\Delta$ varied from 0 to ${\Delta }_{{\rm{m}}{\rm{ax}}}/2\pi =400$ kHz, and then the system evolves to a nearly steady state in the second stroke due to the increase of the decay rate with a large detuning $\Delta /2\pi =400$ kHz. In the third stroke, the LZS process occurs, resulting in population oscillation. After the fourth stroke, the system evolves to another nearly steady state as a result of the large detuning $\triangle /2\pi =-400$ kHz and slow-varying dissipation. Finally, a Landau-Zener transition occurs again in the fifth stroke with the detuning $\Delta$ tuned from ${\Delta }_{\min }/2\pi =-400$ kHz to 0, resulting in the final state $\left|{\psi }_{-}\right\rangle =(\left|e\right\rangle -\left|g\right\rangle )/\sqrt{2}$ ^49,50^. In contrast, for the clockwise loop from $\left|{\psi }_{+}\right\rangle =(\left|e\right\rangle +\left|g\right\rangle )/\sqrt{2}$, the system evolves to a steady state after the fourth stroke and experiences a LZS with the detuning $\Delta$ tuned from ${\Delta }_{\max }/2\pi =400$ kHz to 0, accumulating a Stückelberg phase^50^ and thus leading to the final state $\left|{\psi }_{+}\right\rangle =(\left|e\right\rangle +\left|g\right\rangle )/\sqrt{2}$.

Since the system evolves along a closed trajectory, we can evaluate the network using ${W}_{1}={\int }_{0}^{t}\rho (t){{\rm{d}}H}(t)$, where $\rho (t)$ describes the state of the two-level system governed by $H\left(t\right)=\Delta (t)\left|e\right\rangle {{\langle }}e{|}$. For the clockwise encirclement in Fig. 2b, since the isochoric strokes produce no work, here we only consider the first, third, and fifth strokes with the first and fifth strokes executing expansion and the third stroke executing compression. Moreover, the bath performs work on the system in the iso-decay compression stroke (third stroke), and the system produces work during the two iso-decay expansion strokes. The mean population in $\left|e\right\rangle$ in the two expansion strokes is higher than that in the compression stroke, implying a positive network, and thus, the system behaves as the QHE. On the contrary, the counterclockwise loop in Fig. 2a leads to a negative network due to the higher mean population in $\left|e\right\rangle$ in the two compression strokes than that in the expansion stroke. This makes the system perform as a QR. Thus, we conclude that the loops that do not result in asymmetric mode conversion (CW loop starting at $\left|{\psi }_{+}\right\rangle$ and CCW loop starting at $\left|{\psi }_{-}\right\rangle$) perform as a QHE (Fig. 2b) or a QR (Fig. 2a).

We mention that Rabi frequency $\Omega$ remains constant in our implementation of QHE and QR cycles, indicating that the working substance keeps interacting with the 729-nm laser. The heating and cooling processes are accomplished by modulating the detuning and/or the decay rate. For example, the working substance reaches the thermal equilibrium with the thermal baths by changing ${\gamma }_{{\rm{eff}}}$ while keeping a constant value of Δ^6,54,55^.

### Chiral dynamics

**Fig. 3:**
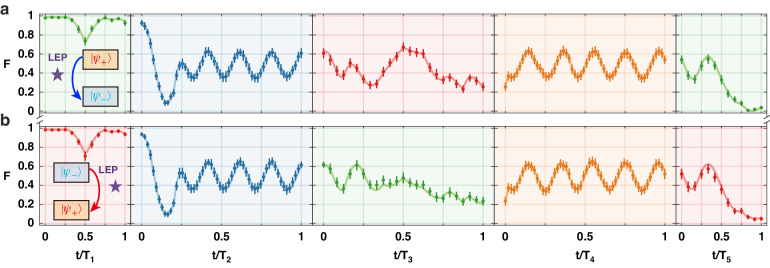
Closed loops that lead to mode conversion. **a** Counterclockwise loop starting at $\left|{\psi }_{+}\right\rangle$ and ending at $\left|{\psi }_{-}\right\rangle$. **b** Clockwise loop starting at $\left|{\psi }_{-}\right\rangle$ and ending at $\left|{\psi }_{+}\right\rangle$. Evolution of the system’s state along the closed loop trajectory is characterized by the fidelity $\langle {\psi }_{+}|\rho (t)\left|{\psi }_{+}\right\rangle$ in (**a**) and the fidelity $\langle {\psi }_{-}|\rho (t)\left|{\psi }_{-}\right\rangle$ in (**b**). The circles and error bars respectively denote the average and standard deviations of 10000 measurements. The solid curves are obtained by simulating master equations. Durations of the five strokes are *T*_1_ = *T*_5_ = 6 μs, *T*_3_ = 12 μs, and *T*_2_ = *T*_4_ = 150 μs. Other parameters are Ω/2*π* = 120 kHz, Δ_min_/2*π* = −400 kHz, Δ_max_/2*π* = 400 kHz, *γ*_min_ ≈ 0 kHz, and *γ*_max_ ≈ 1.45 MHz

The chiral behavior and asymmetric mode conversion appear for counterclockwise loop starting at $\left|{\psi }_{+}\right\rangle$ and the clockwise loop starting at $\left|{\psi }_{-}\right\rangle$, as witnessed in Fig. 3. In both of these two cases, the system cannot return to the starting state after the loop is completed. Together with the observation depicted in Fig. 2a, we conclude that the final states (i.e., the endpoints of the loops) depend only on the encircling direction, not on the initial state (i.e., the starting point of the loops): A CW (or CCW) loop in the vicinity of the LEP but not encircling it ends up at the final state $\left|{\psi }_{+}\right\rangle$ (or $\left|{\psi }_{-}\right\rangle$) regardless of whether the initial state is $\left|{\psi }_{+}\right\rangle$ or $\left|{\psi }_{-}\right\rangle$.

It is evident that whether the system evolves back to the starting point or not is essentially associated with the LZS process and the breakdown of the adiabaticity when executing the closed-loop trajectory. This is reflected in the fifth stroke of the loop when a state transfer occurs after experiencing a Landau–Zener transition (Figs. 2 and 3). For the clockwise encirclement starting from $\left|{\psi }_{+}\right\rangle$ or the counterclockwise encirclement from $\left|{\psi }_{-}\right\rangle$, the Landau–Zener transition in the fifth stroke determines if the closed thermodynamic cycle works as the chiral QHE or QR. In contrast, for the clockwise loop starting at $\left|{\psi }_{-}\right\rangle$ or the counterclockwise loop at $\left|{\psi }_{+}\right\rangle$, this Landau–Zener transition determines the chiral behavior. In other words, when the system starts at the state $\left|{\psi }_{+}\right\rangle$, a clockwise loop in the vicinity of (not encircling) the LEP ends in the same state, whereas a counterclockwise loop ends at the orthogonal state $\left|{\psi }_{-}\right\rangle$. So, the system acts as a QHE for the clockwise loop and as a mode converter for the counterclockwise loop. Similarly, when the system starts at the state $\left|{\psi }_{-}\right\rangle$, a clockwise loop in the vicinity of (not encircling) the LEP ends in the orthogonal state $\left|{\psi }_{+}\right\rangle$ whereas a counterclockwise loop ends at the same state $\left|{\psi }_{-}\right\rangle$. So, the system acts as a QR for the counterclockwise loop and as a mode converter for the clockwise loop.

Here we emphasize that, the LZ process in the fifth stroke is the most essential to the chiral behavior and asymmetric mode conversion. When the evolution time of the fifth stroke is too short, our system works in the non-adiabatic regime and lacks sufficient time to follow the variation of the detuning, resulting in non-adiabatic transitions between two eigenstates via phase accumulation through the LZS process^48–50^. These transitions would prevent our system from exhibiting chiral behavior and asymmetric mode conversion. In contrast, when the evolution time of the fifth stroke is too long, our system works in the adiabatic regime and smoothly follows the variation of the detuning. Nevertheless, the evolution duration is restricted by the decoherence time, as clarified by the numerical simulation results shown in Fig. S8.

Although the displayed loops exclude the LEP, our numerical simulations suggest a strong relevance of the observed chiral behavior to the existence of the LEP. If the loop is too far away from the LEP, i.e., ${\gamma }_{{\rm{eff}}}$ is too small, no chirality would appear (see Supplementary Materials). However, if the system parameters are chosen such that the loops are closer to the LEP but not encircling it, the populations of $\left|{\psi }_{+}\right\rangle$ and $\left|{\psi }_{-}\right\rangle$ interchange for the clockwise loop starting at $\left|{\psi }_{-}\right\rangle$ and the counterclockwise loop starting at $\left|{\psi }_{+}\right\rangle$ (see Supplementary Materials). Moreover, since the loops do not encircle the LEP, no topological phase transition is involved in the system’s response. We note that an essential ingredient for the observed chirality is the transition of the system between two Riemann sheets when the parameters are tuned to form a closed loop. Considering the system state evolves only within a single Riemann sheet, we see the encircling direction determines whether the chirality exists or not. For example, Figs. S5 and S6 in Supplementary Materials show that chiral behavior appears only in the counterclockwise and clockwise encirclements, respectively. Otherwise the final state neither returns to the initial state nor has a state convention between $\left|{\psi }_{+}\right\rangle$ and $\left|{\psi }_{-}\right\rangle$. These phenomena can be called non-reciprocal chirality, i.e., unidirectional chirality.

As our chiral behavior and asymmetric mode conversion result from the LEP rather than the Hamiltonian EP^34–41,56,57,58^, we have fully captured the quantum dynamics, including the quantum jumps and associated noises, significantly extending the realization conditions of chiral behavior and asymmetric mode conversion, and considerably reducing the experimental difficulties in quantum control and measurement. Realizing chiral behavior without encircling an LEP helps reduce the parameter space needed to steer the system. For example, encircling the LEP required varying ${\gamma }_{\max }$ to values >4$\Omega$^33^${\rm{;}}$ ; however, observing chiral behavior without encircling the LEP requires varying ${\gamma }_{\max }$ to values <2$\Omega$. This helps keep our system in the quantum regime for achieving the chiral behavior and asymmetric mode conversion and also opens up possibilities for a more focused and extended exploration of the physical properties associated with the LEP. Moreover, the combination of the adiabaticity breakdown^38^ and the Landau–Zener–Stückelberg phase^49,50^ leads to the presented chirality, whose physical mechanics is distinct from the one resulting from spontaneous chiral symmetry breaking^59,60^. Furthermore, our observed chiral behavior and asymmetric mode conversion strongly depend on the LEPs resulting from quantum jumps. Characterized by quantum jumps, the above phenomena in our experimental system are inherently quantum. In contrast, without quantum jumps, our experimental system would return to Hamiltonian EPs^25,61^, whose chiral behavior and asymmetric mode conversion have been theoretically predicted^42^ and experimentally observed with waveguides^44^ and fibers^45^.

## Discussion

In this work, we have experimentally demonstrated, for the first time, a chiral behavior in a single trapped-ion system without dynamically encircling its LEP. We show clearly that asymmetric mode conversion is directly related to the topological landscape of the Riemann surfaces and not necessarily to encircling an LEP of this quantum system, supporting previous reports for classical systems. Our experiments may open up new avenues in understanding the chiral and topological behaviors in non-Hermitian systems and bridging chirality and quantum thermodynamics.

## Materials and methods

### Thermodynamic quantities

We present here the definitions of the thermodynamic quantities, which are the network ${\rm{W}}$ and the efficiency ${\rm{\eta }}$. For our trapped-ion system, we define the internal energy of the quantum heat engine as ${\rm{U}}={\rm{t}}{\rm{r}}({\rm{\rho }}{\rm{H}})$, where ρ and H are the density matrix and Hamiltonian of the quantum system, respectively. In our experiments, the Hamiltonian of the working substance is given by ${{\rm{H}}=\Delta }_{e}\left|e\right\rangle \langle {e|}$. The coupling strength Ω, and the effective dissipation rate ${{\rm{\gamma }}}_{{eff}}$ are employed to tune the system such that the QHE cycle and the QR cycle are performed, as explained in the main text.

In classical thermodynamics, the first law of thermodynamics is expressed as d*U* = d*W* + d*Q*. In contrast, in quantum thermodynamics, it is expressed as

$${\rm{d}}U={\rm{d}}{H{\rm {tr}}}\left({\rm{\rho }}{H}\right)={\rm{tr}}\left(H{\rm{d}}{\rho }\right)+{\rm{tr}}\left({\rho }{\rm{d}}H\right)$$

where the work and heat in the differential form are defined as ${\rm{d}W}={\rho }{\rm{d}H}$ and ${\rm{d}}Q=H{\rm{d}}{\rm{\rho }}$, respectively. Then, the work ${{W}}_{{{\rm {in}}}}$ done by the environment and the one ${{W}}_{{\rm {{out}}}}$ by the working substance are given by ${{W}}_{{\rm {{in}}}}=-\sum _{i}{{\rm{\rho }}}_{i}{\rm{d}}{H}_{i}$ (for ${\rm{d}}{H}_{i}\, > \,0$) and ${{W}}_{{\rm {{out}}}}=-\sum _{i}{{\rm{\rho }}}_{i}{\rm{d}}{H}_{i}$ (for ${\rm{d}}{H}_{i} \,< \,0$), respectively. As discussed in the main text, ${{W}}_{{{\rm {in}}}}$ and ${{W}}_{{\rm {{out}}}}$ are calculated, respectively, in the iso-decay compression and iso-decay expansion strokes of our trapped-ion QHE and QR cycles. Thus, the network acquired can be written as

$${{W}}_{{\rm {{net}}}}={{W}}_{{\rm {{in}}}}+{{W}}_{{\rm {{out}}}}=-\sum _{i}{{\rm{\rho }}}_{i}{\rm{d}}{H}_{i}$$

which is used to calculate the network in Figs. 2 and 3.

### Supplementary information

Supplementary Information for

## Data Availability

The data illustrated in the figures within this paper are available from the corresponding authors upon request.

## Code availability

The custom codes used to generate the results presented in this paper are available from the corresponding authors upon request.
