# Supplementary material for: Chiral quantum heating and cooling with an optically controlled ion
Source: Light Sci Appl. 2024 Jun 26;13:143. doi: 10.1038/s41377-024-01483-5 (PMC11199633; doi:10.1038/s41377-024-01483-5)
Supplement: Supplementary file 1 — Supplementary Information for [file 41377_2024_1483_MOESM1_ESM.pdf]

Supplementary Information for "Chiral quantum heating and cooling with an optically controlled ion"

J.-T. Bu,[1,2,*] J.-Q. Zhang,[1,*] G.-Y. Ding,[1,2,*] J.-C. Li,[1,2] J.-W. Zhang,[3] B. Wang,[1,2] W.-Q. Ding,[1,2] W.-F. Yuan,[1,2] L. Chen,[1,3] Q. Zhong,[4] A. Kecebas,[4] Ş. K. Özdemir,[4,†] F. Zhou,[1,3,‡] H. Jing,[5,§] M. Feng[1,3,6,¶]

[1] *State Key Laboratory of Magnetic Resonance and Atomic and Molecular Physics, Wuhan Institute of Physics and Mathematics, Innovation Academy of Precision Measurement Science and Technology, Chinese Academy of Sciences, Wuhan 430071, China*

[2] *University of the Chinese Academy of Sciences, Beijing 100049, China*

[3] *Research Center for Quantum Precision Measurement, Guangzhou Institute of Industry Technology, Guangzhou, 511458, China*

[4] *Department of Engineering Science and Mechanics, and Materials Research Institute, Pennsylvania State University, University Park, State College, Pennsylvania 16802, USA*

[5] *Key Laboratory of Low-Dimensional Quantum Structures and Quantum Control of Ministry of Education, Department of Physics and Synergetic Innovation Center for Quantum Effects and Applications, Hunan Normal University, Changsha 410081, China*

[6] *Department of Physics, Zhejiang Normal University, Jinhua 321004, China*

We present details about the dynamics and chirality of quantum heat engine (QHE) and quantum refrigerator (QR). We also discuss the encirclement within single Riemann sheets, i.e., in half of the encirclement area as considered in the main text.

* Co-first authors with equal contribution

† sko9@psu.edu

‡ zhoufei@wipm.ac.cn

§ jinghui73@foxmail.com

¶ mangfeng@wipm.ac.cn

I. order parameter with respect to Liouvillian exceptional point

Our system is governed by the Lindblad master equation

$$\dot{\rho}(t) = -i[H_{eff}, \rho] + \frac{\gamma_{eff}}{2}(2\sigma_-\rho\sigma_+ - \sigma_+\sigma_-\rho - \rho\sigma_+\sigma_-) = \mathcal{L}\rho \tag{1}$$

where $\mathcal{L}$ is called Liouvillian superoperator and ρ is the density operator of the system. Here the density matrix ρ is expressed by $\begin{pmatrix} \rho_{ee} & \rho_{eg} \\ \rho_{ge} & \rho_{gg} \end{pmatrix}$, with the raising and lowering operators defined as $\sigma_+ = \begin{pmatrix} 0 & 1 \\ 0 & 0 \end{pmatrix}$ and $\sigma_- = \begin{pmatrix} 0 & 0 \\ 1 & 0 \end{pmatrix}$, respectively. According to the corresponding equations in the main text, we can write $\mathcal{L}$ as

$$\mathcal{L} = \begin{pmatrix} -\gamma_{eff} & i\Omega/2 & -i\Omega/2 & 0 \\ i\Omega/2 & -(\gamma_{eff}/2 + i\Delta) & 0 & -i\Omega/2 \\ -i\Omega/2 & 0 & -(\gamma_{eff}/2 - i\Delta) & i\Omega/2 \\ \gamma_{eff} & -i\Omega/2 & i\Omega/2 & 0 \end{pmatrix} \tag{2}$$

By setting $\Delta = 0$, we find the eigenvalues of $\mathcal{L}$ as $\lambda_1 = 0$, $\lambda_2 = -\gamma_{eff}/2$, $\lambda_3 = \frac{1}{4}(-3\gamma_{eff} - \sqrt{\gamma_{eff}^2 - 16\Omega^2})$, and $\lambda_4 = \frac{1}{4}(-3\gamma_{eff} + \sqrt{\gamma_{eff}^2 - 16\Omega^2})$. It is evident that the eigenvalues λ_3 and λ_4 becomes degenerate at $\gamma_{eff} = 4\Omega$, which corresponds to the Liouvillian exceptional point (LEP). For $\gamma_{eff} > 4\Omega$, we have real λ_3 and λ_4 with a splitting of $|\lambda_4 - \lambda_3| = \xi/2$ with $\xi = \sqrt{\gamma_{eff}^2 - 16\Omega^2}$. For $\gamma_{eff} < 4\Omega$, on the other hand, λ_3 and λ_4 form a complex conjugate pair with a splitting of $|\xi|/2$ in their imaginary parts.

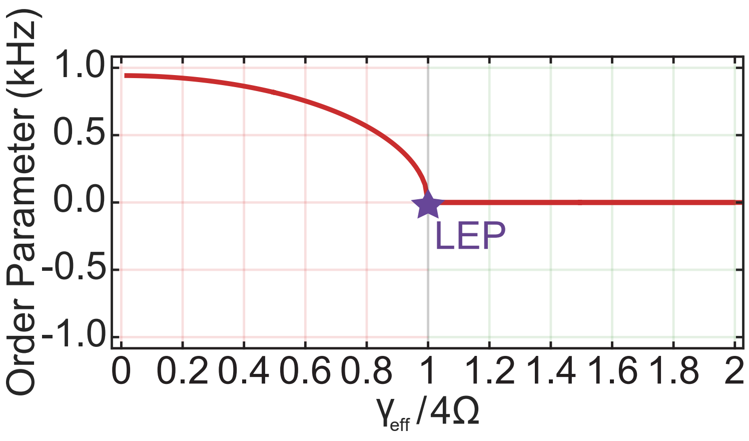

Fig.S. 1. Topological phase transition with respect to the Liouvillian exceptional point (LEP) that is determined by the decay rate γ_{eff} and the drive strength Ω.

The topological phase transition occurs at the LEP, as shown in Fig.S. 1. Since the LEP is excluded in our displayed encirclements, no topological phase transition occurs in our experiment. Nevertheless, the influence from the LEP is witnessed in our experiment. As shown in the following sections, we have observed experimentally the strong relevance of the chirality to the LEP and the associated Riemann sheets.

In contrast, with the quantum jumps ignored, the above master equation (1) is reduced to

$$\dot{\rho}(t) = -i[H_{eff}, \rho] - \frac{\gamma_{eff}}{2}(\sigma_+\sigma_-\rho + \rho\sigma_+\sigma_-) = -i(H_{NH}\rho - \rho H_{NH}^\dagger) = \mathcal{L}_H\rho \tag{3}$$

where $H_{NH} = H_{eff} - i\frac{\gamma_{eff}}{2}\sigma_+\sigma_-$ is the non-Hermitian Hamiltonian and

$$\mathcal{L}_H = \begin{pmatrix} -\gamma_{eff} & i\Omega/2 & -i\Omega/2 & 0 \\ i\Omega/2 & -(\gamma_{eff}/2 + i\Delta) & 0 & -i\Omega/2 \\ -i\Omega/2 & 0 & -(\gamma_{eff}/2 - i\Delta) & i\Omega/2 \\ 0 & -i\Omega/2 & i\Omega/2 & 0 \end{pmatrix} \tag{4}$$

is the Liouvillian operator without quantum jumps. Both H_{NH} and $\mathcal{L}_H$ have been utilized to experimentally verify the phase transition of EP for $\Delta = 0$ kHz [1]. The above derivations illustrate, without involving the quantum jump item $\sigma_-\rho\sigma_+$, the LEP returns to the Hamiltonian EP (HEP). In other words, the HEP cannot describe the entire dynamics of the open quantum system.

We find the eigenvalues of $\mathcal{L}_H$ as $\tilde{\lambda}_1 = \tilde{\lambda}_2 = -\gamma_{eff}/2$, $\tilde{\lambda}_3 = (-\gamma_{eff} - \sqrt{\gamma_{eff}^2 - 4\Omega^2})/2$ and $\tilde{\lambda}_4 = (-\gamma_{eff} + \sqrt{\gamma_{eff}^2 - 4\Omega^2})/2$. The eigenvalues $\tilde{\lambda}_3$ and $\tilde{\lambda}_4$ become degenerate at $\gamma_{eff} = 2\Omega$, corresponding to the HEP. Real $\tilde{\lambda}_3$ and $\tilde{\lambda}_4$ present a splitting of $|\tilde{\lambda}_3 - \tilde{\lambda}_4| = \sqrt{\gamma_{eff}^2 - 4\Omega^2}$ when $\gamma_{eff} > 2\Omega$, while $\tilde{\lambda}_3$ and $\tilde{\lambda}_4$ become a complex conjugate pair with a splitting of $\sqrt{\gamma_{eff}^2 - 4\Omega^2}$ in their imaginary parts.

Considering the experimental parameters and the presence of quantum jumps, we conclude that the EP observed in our system is an LEP rather than a HEP. LEP and HEP have been observed in previous experiments [4] and [5], respectively. These experiments illustrate that, when the decay rate is fixed, the coupling strength for the LEP [4] is much smaller than that for the HEP [5], and this difference results from quantum jumps. Additionally, in the case of $\gamma_{eff} = 0$ kHz, the eigenenergies of the LEP and HEP are identical, and we can obtain $\lambda_1 = \lambda_2 = \tilde{\lambda}_2 = \tilde{\lambda}_1 = 0$, $\lambda_3 = \tilde{\lambda}_3 = i\Omega$, and $\lambda_4 = \tilde{\lambda}_4 = -i\Omega$ for the identical Lindblad operators (2) and (4). In this case, the dynamics of the LEP and HEP share identical results

with the same initial states.

Besides, in our experiments, the Rabi frequency is $\Omega/2\pi = 120$ kHz. Based on other experimental parameters, we have theoretically calculated the decay rate for the LEP as $\gamma_{\mathrm{LEP}} = 4\Omega \approx 3.0$ MHz, and for the HEP as $\gamma_{\mathrm{HEP}} = 2\Omega \approx 1.5$ MHz. None of these decay rates is smaller than the maximum decay rate $\gamma_{\max} \approx 1.45$ MHz in our experiment. As a result, although there is no HEP in our experiments, we can say, neither the HEPs nor the LEPs are encircled in our experimental loops (see Fig.S. 2).

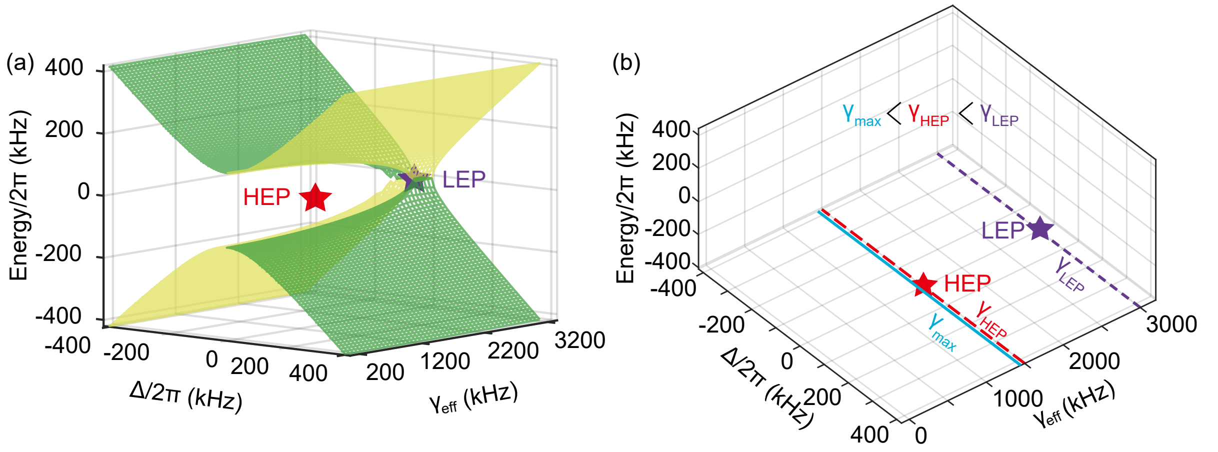

Fig.S. 2. (a) Positions of the LEP and HEP in the parameter space for the eigenenergy Riemann surface. It shows, on the eigenenergy Riemann surface, the EPs presented correspond to the LEPs rather than the HEPs. (b) Projected decay rates ($\gamma_{\max}$, γ_{HEP}, and γ_{LEP}) in the $\Delta - \gamma_{eff}$ plane with the Rabi frequency of $\Omega = 120$ kHz.

II. Dynamics of quantum heat engine and quantum refrigerator cycles

We execute the clockwise and counterclockwise encirclements without encircling the LEPs as designed in the main text by elaborately controlling the 854 nm laser (for tuning γ_{eff}) and the 729 nm laser (for tuning Δ) with $\Omega/2\pi \approx 120$ kHz in the whole process. The dynamical processes of the four encirclements are demonstrated by observing the evolution of the fidelity $\langle\psi_+|\rho(t)|\psi_+\rangle$ or $\langle\psi_-|\rho(t)|\psi_-\rangle$ as shown in Fig. S 3(a-d).

For the clockwise QHE cycle in Fig.S. 3(c), we first prepare the state of the system in $|g\rangle$ and then apply a $\pi/2$ pulse to prepare the initial state $|\psi_+\rangle = (|e\rangle + |g\rangle)/\sqrt{2}$, corresponding to $\rho_A = \left(\begin{smallmatrix} 0.4925 & 0.4925 \\ 0.4925 & 0.4925 \end{smallmatrix}\right)$ experimentally (due to the imperfect ground state preparation and subsequent $\pi/2$ pulse operation), and thus the initial fidelity is $\langle\psi_+|\rho_A|\psi_+\rangle = 0.985$. Then we execute the first iso-decay expansion process by decreasing the detuning Δ from $\Delta = 0$

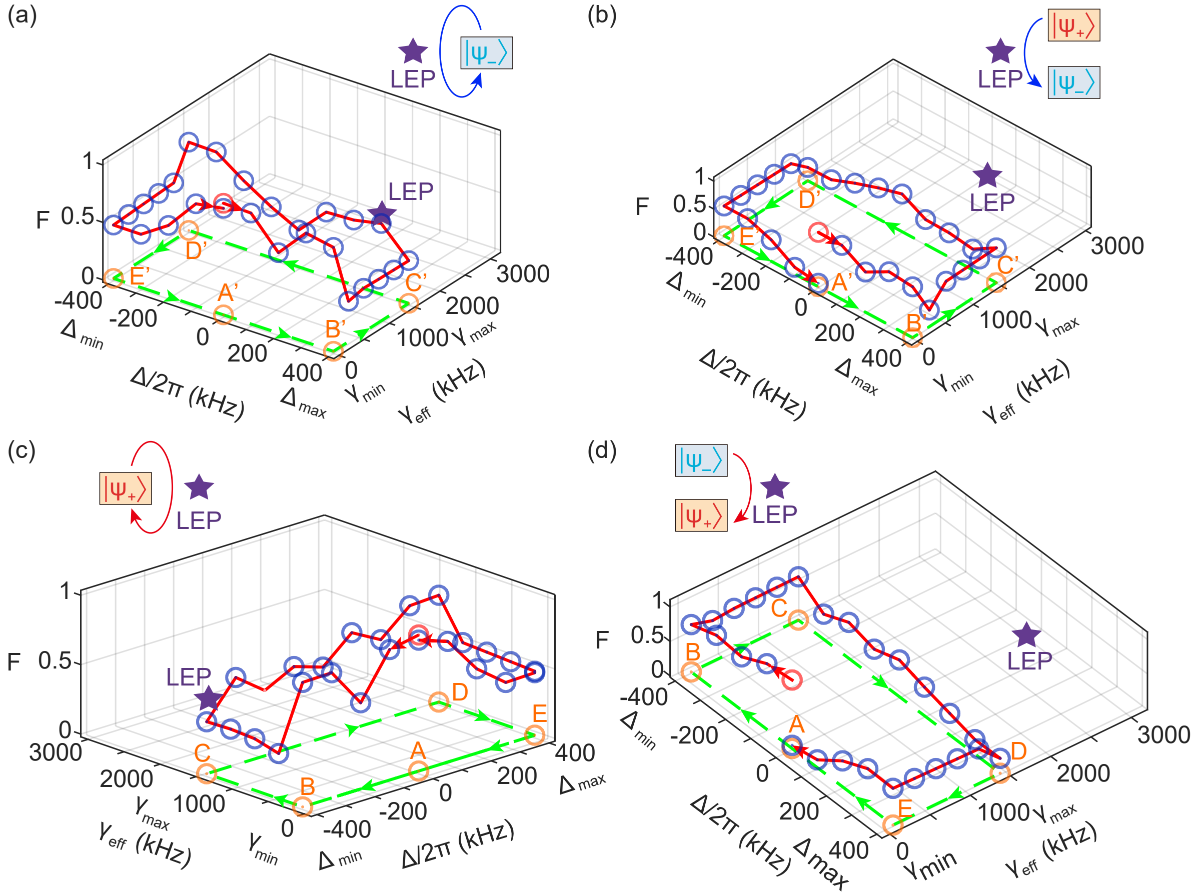

Fig.S. 3. Variation of fidelity with respect to the detuning and the effective decay rate γ_{eff}, where we consider the fidelity $\langle\psi_+|\rho(t)|\psi_+\rangle$ or $\langle\psi_-|\rho(t)|\psi_-\rangle$ when completing a clockwise or a counterclockwise encirclement, employing $\Delta_{\min}/2\pi = -400$ kHz, $\Delta_{\max}/2\pi = 400$ kHz, $\gamma_{\min} \approx 0$ kHz, and $\gamma_{\max} \approx 1.45$ MHz. The red dots represent the starting points and the red lines show the evolution trends, instead of the real evolution trajectories, with the red arrows denoting the encircling directions. The green dashed curves are the projection of the solid red curves on the bottom plane for guiding eyes. Five orange corner points A, B, C, D, E (A', B', C', D', E') are labeled for convenience of description in the text. The LEPs are labeled by the purple stars.

kHz to $\Delta_{\min} = -2\pi \times 400$ kHz while $\gamma_{eff} \approx 0$ kHz remains unchanged with $T_1 = 6$ μs. The system evolves to $\rho_B = \begin{pmatrix} 0.668 & 0.444-0.122i \\ 0.444+0.122i & 0.317 \end{pmatrix}$ as numerically calculated. Then we carry out the isochoric cooling process by increasing the decay rate γ_{eff} from $\gamma_{\min} \approx 0$ kHz to $\gamma_{\max} \approx 1.43$ MHz while $\Delta = \Delta_{\min}$ is fixed. Experimentally, we separate the evolution into five steps with the increasing decay rate, i.e. $T_{2-1} = 30$ μs, $\gamma_{eff} \approx 0$ kHz; $T_{2-2} = 30$ μs, $\gamma_{eff} \approx 286$ kHz; $T_{2-3} = 30$ μs, $\gamma_{eff} \approx 572$ kHz; $T_{2-4} = 30$ μs, $\gamma_{eff} \approx 858$ kHz; $T_{2-5} = 30$ μs, $\gamma_{eff} \approx 1.14$ MHz, the system evolves to $\rho_C = \begin{pmatrix} 0.021 & -0.107-0.089i \\ -0.107+0.089i & 0.964 \end{pmatrix}$ after this process due to the large decay and detuning. The next stroke is the iso-decay compression with the detuning tuned from $\Delta_{\min}$ to $\Delta_{\max}$ and the constant value of $\gamma_{eff} = \gamma_{\max}$, the duration of this stroke is $T_3 = 12$ μs, and thus the system evolves to $\rho_D = \begin{pmatrix} 0.104 & 0.284-0.008i \\ 0.284+0.008i & 0.88 \end{pmatrix}$. The

fourth step is the isochoric heating process with the decay rate γ_{eff} tuned from $\gamma_{\max} \approx 1.43$ MHz to $\gamma_{\min} \approx 0$ kHz when $\Delta = \Delta_{\max}$ is fixed. During this process, our experimental operation is to separate the evolution into five steps along with the decay, i.e., $T_{4-1} = 30$ μs, $\gamma_{eff} \approx 1.43$ MHz; $T_{4-2} = 30$ μs, $\gamma_{eff} \approx 1.14$ MHz; $T_{4-3} = 30$ μs, $\gamma_{eff} \approx 858$ kHz; $T_{4-4} = 30$ μs, $\gamma_{eff} \approx 572$ kHz; $T_{4-5} = 30$ μs, $\gamma_{eff} \approx 286$ kHz, the system evolves to a steady state $\rho_E = \left(\begin{smallmatrix} 0.021 & -0.036-0.137i \\ -0.036+0.137i & 0.964 \end{smallmatrix}\right)$ after this stroke with negligible non-diagonal elements due to the large decay and the long duration time. The final step is the iso-decay expansion stroke process by decreasing the detuning from $\Delta = 2\pi \times 400$ kHz to $\Delta = 0$ kHz while $\gamma_{eff} = \gamma_{\min} \approx 0$ kHz remains unchanged with $T_5 = 6$ μs, and thus the system experiences a Landau-Zener transition and accumulate a Stückelberg phase, reaching the final state $\left(\begin{smallmatrix} 0.651 & 0.464-0.044i \\ 0.464+0.044i & 0.334 \end{smallmatrix}\right)$. The calculated final fidelity is $\langle\psi_+|\rho(t)|\psi_+\rangle = 0.957$, indicating that the system finally returns to the initial state after the encirclement.

By contrast, for the counterclockwise QR cycle in Fig.S. 3(a), we prepare the initial state $|\psi_0\rangle = |\psi_-\rangle = (|e\rangle - |g\rangle)/\sqrt{2}$, corresponding to $\rho_{A'} = \left(\begin{smallmatrix} 0.4925 & -0.4925 \\ -0.4925 & 0.4925 \end{smallmatrix}\right)$ experimentally, and the initial fidelity is $\langle\psi_-|\rho(A')|\psi_-\rangle = 0.985$. We first execute the iso-decay compression stroke by increasing the detuning Δ from $\Delta = 0$ kHz to $\Delta_{\max} = 2\pi \times 400$ kHz while $\gamma_{eff} \approx 0$ kHz remains unchanged with $T_1 = 6$ μs, and thus the system evolves to $\rho_{B'} = \left(\begin{smallmatrix} 0.668 & -0.444+0.122i \\ -0.444-0.122i & 0.317 \end{smallmatrix}\right)$. Then we carry out the isochoric cooling stroke by increasing the decay rate γ_{eff} from $\gamma_{\min} \approx 0$ kHz to $\gamma_{\max} = 1.43$ MHz while $\Delta = \Delta_{\max}$ is fixed. Experimentally, we separate the evolution into five steps with the increasing decay rate, i.e. $T_{2-1} = 30$ μs, $\gamma_{eff} \approx 0$ kHz; $T_{2-2} = 30$ μs, $\gamma_{eff} \approx 286$ kHz; $T_{2-3} = 30$ μs, $\gamma_{eff} \approx 572$ kHz; $T_{2-4} = 30$ μs, $\gamma_{eff} \approx 858$ kHz; $T_{2-5} = 30$ μs, $\gamma_{eff} \approx 1.14$ MHz, and thus the system evolves to $\rho_{C'} = \left(\begin{smallmatrix} 0.021 & 0.128-0.055i \\ -0.128+0.055i & 0.964 \end{smallmatrix}\right)$ after this stroke. The next stroke is the iso-decay expansion with the detuning tuned from $\Delta_{\max}$ to $\Delta_{\min}$ with the constant value of $\gamma_{eff} = \gamma_{\max}$ with $T_3 = 12$ μs, and thus the system evolves to $\rho_{D'} = \left(\begin{smallmatrix} 0.098 & -0.112-0.26i \\ -0.112+0.26i & 0.886 \end{smallmatrix}\right)$ after this stroke. The fourth step is the isochoric heating process with decay rate γ_{eff} tuned from $\gamma_{\max} \approx 1.43$ MHz to $\gamma_{\min} \approx 0$ kHz when $\Delta = \Delta_{\min}$ is fixed. During this process, we divide the evolution into five stages of the decay rate, i.e., $T_{4-1} = 30$ μs, $\gamma_{eff} \approx 1.43$ MHz; $T_{4-2} = 30$ μs, $\gamma_{eff} \approx 1.14$ MHz; $T_{4-3} = 30$ μs, $\gamma_{eff} \approx 858$ kHz; $T_{4-4} = 30$ μs, $\gamma_{eff} \approx 572$ kHz; $T_{4-5} = 30$ μs, $\gamma_{eff} \approx 286$ kHz, and thus the system evolves to a steady state $\rho_{E'} = \left(\begin{smallmatrix} 0.021 & 0.036-0.137i \\ 0.037+0.137i & 0.964 \end{smallmatrix}\right)$ after this stroke with negligible non-diagonal elements. The final step is the iso-decay compression stroke by increasing the detuning from $\Delta = -2\pi \times 400$ kHz to $\Delta = 0$ kHz while $\gamma_{eff} = \gamma_{\min} \approx 0$ kHz remains unchanged with $T_5 = 6$ μs. The system experiences a LZS

process and accumulates a Stückelberg phase contrary to the clockwise encirclements, thus reaching the final state $\begin{pmatrix} 0.651 & -0.464-0.044i \\ -0.464+0.044i & 0.334 \end{pmatrix}$. The final fidelity is $\langle\psi_-|\rho(t)|\psi_-\rangle = 0.957$, indicating that the system almost evolves to the initial state at the end of the encirclement.

While for the clockwise encirclement depicted in Fig.S. 3(d), we prepare the state initially in $|\psi_0\rangle = |\psi_-\rangle = (|e\rangle - |g\rangle)/\sqrt{2}$, corresponding to $\rho_A = \begin{pmatrix} 0.4925 & -0.4925 \\ -0.4925 & 0.4925 \end{pmatrix}$ experimentally, the fidelity is initially $\langle\psi_-|\rho(A)|\psi_-\rangle = 0.985$. We repeat the experimental sequence of the QHE cycle, then the system evolves to a steady state $\rho_E = \begin{pmatrix} 0.021 & -0.036-0.137i \\ -0.036+0.137i & 0.964 \end{pmatrix}$ after the fourth stroke (the same as the QHE cycle). After experiencing a LZS in the final stroke, the system evolves to $\begin{pmatrix} 0.453 & 0.445+0.206i \\ 0.445-0.206i & 0.532 \end{pmatrix}$. The final fidelity is $\langle\psi_-|\rho(t)|\psi_-\rangle = 0.0487$, showing the system cannot return to the initial state after the encirclement. For the counterclockwise encirclement depicted in Fig.S. 3(b), we prepare the initial state $|\psi_0\rangle = |\psi_+\rangle = (|e\rangle + |g\rangle)/\sqrt{2}$, corresponding to $\rho_{A'} = \begin{pmatrix} 0.4925 & 0.4925 \\ 0.4925 & 0.4925 \end{pmatrix}$ experimentally, the initial fidelity is $\langle\psi_+|\rho(A')|\psi_+\rangle = 0.985$. We repeat the experimental sequence of the QR cycle, then the system evolves to a steady state $\rho_{E'} = \begin{pmatrix} 0.021 & 0.058-0.129i \\ 0.058+0.129i & 0.964 \end{pmatrix}$ after the fourth stroke (the same as the QR cycle). After the fifth stroke, the system evolves to $\begin{pmatrix} 0.667 & -0.454-0.075i \\ -0.454+0.075i & 0.318 \end{pmatrix}$. The final fidelity is $\langle\psi_+|\rho(t)|\psi_+\rangle = 0.039$, showing that the system cannot evolve back to the initial state after the encirclement.

As a consequence, we conclude that the chirality depends on both the initial state and the encircling direction, which results in different thermodynamic processes, i.e., either QHE or QR.

III. State evolution with respect to eigenstates

We numerically calculate the state evolutions with respect to the eigenstates of the time-dependent effective Hamiltonian $H_{eff} = (\Delta - i\gamma_{eff})|e\rangle\langle e| + \Omega/2(|e\rangle\langle g| + |g\rangle\langle e|)$. The encirclements plotted in Figs.S. 4(a)-(d) correspond to the trajectories depicted in Figs. 1(c)-(f) in the main text, respectively.

IV. Variation of the chirality when approaching LEP

In this section, we carry out numerical simulation for the fidelity after completing the clockwise or counterclockwise encirclements. Considering the initial states $|\psi_+\rangle$ and $|\psi_-\rangle$, respectively, we sweep $\gamma_{\max}$ but fix other parameters, monitoring how the population of the

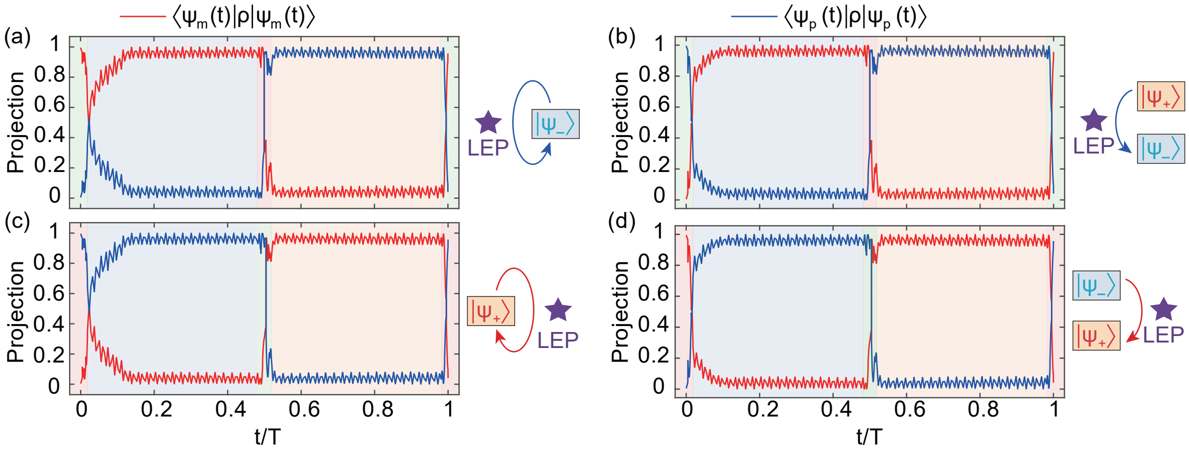

Fig.S. 4. State evolution with respect to the eigenstates of the effective Hamiltonian. The evolution of the encircling state for clockwise (counterclockwise) loops starting from $|\psi_+\rangle$ ($|\psi_-\rangle$) is characterized by the projection $\langle\psi_m(t)|\rho|\psi_m(t)\rangle$ ($\langle\psi_p(t)|\rho|\psi_p(t)\rangle$), where $\psi_p(t)$ and $\psi_m(t)$ correspond, respectively, to the upper and lower branches of the Riemann surface.

final state varies versus $\gamma_{\max}$.

We find a transition from no chirality to chirality with the increasing decay rate $\gamma_{\max}$. When the decay rate $\gamma_{\max}$ is small enough, the Landau-Zener–Stückelberg (LZS) process ensures the final state to be the same as the initial state and no asymmetric mode conversion occurs. As plotted in Fig.S. 5, we see that both the clockwise and counterclockwise encirclements starting from $|\psi_+\rangle$ evolve to the same final state $|\psi_+\rangle$, and the system finally reaches $|\psi_-\rangle$ when starting from $|\psi_-\rangle$. With the increase of the decay rate $\gamma_{\max}$, however, we see asymmetric mode conversion occurring as $\gamma_{\max}/4\Omega \geq 0.05$. This is due to the non-adiabatic transition induced by the enhanced decay. As a result, the chirality appears. Taking the initial state $|\psi_+\rangle$ as an example, we find the non-adiabatic transition leading to a steady state after the fourth stroke when encircling along the clockwise direction. Then an LZS process results in the final state $|\psi_-\rangle$. When a counterclockwise encirclement is performed, the state evolves back to $|\psi_+\rangle$ ultimately. As a result, the decay rate $\gamma_{\max}$ plays a key role in our experiment. Combined with the Landau-Zener transition, it leads to non-adiabatic transitions, resulting in chiral behavior and asymmetric mode conversion (see the starting points for $\gamma_{\max} = 0$ in Fig.S. 5).

We consider that it is possible to have an analytical estimate of the critical decay rate for the transition, although writing a specifically analytical relation of the chirality transition with the decay rate is challenging. The simplest way for such an estimate is to consider the shortest time required for accomplishing a chirality transition. To this end, we assume that

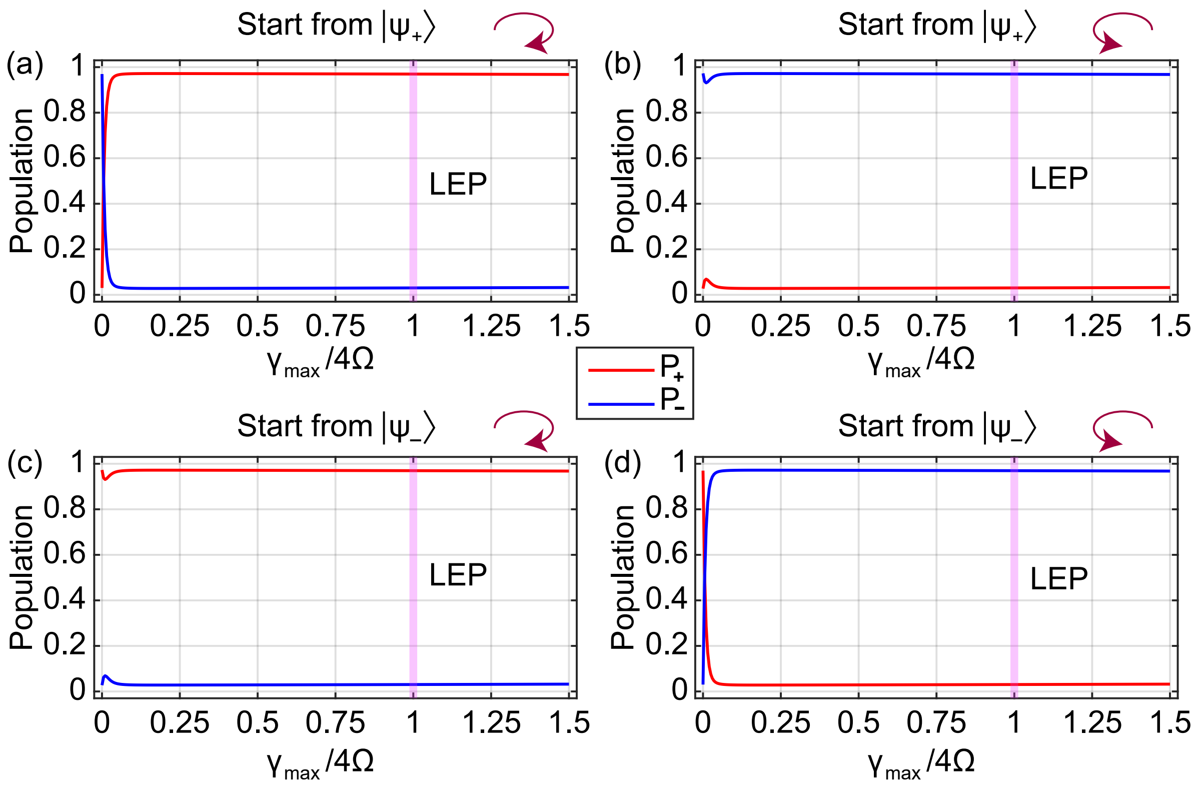

Fig.S. 5. Populations of the final states with respect to $\gamma_{\max}/4\Omega$ in different cases of the initial state and encircling direction. Calculations are made by following parameter values: $\Delta_{\min}/2\pi = -400$ kHz, $\Delta_{\max}/2\pi = 400$ kHz, $\gamma_{\min} = 0$ kHz, $T_1 = T_5 = 6$ μs, $T_2 = T_4 = 50$ μs, $T_3 = 12$ μs. Red and blue solid curves represent the populations of $|\psi_+\rangle$ and $|\psi_-\rangle$, respectively. Pink lines mark the positions of LEPs.

the dissipation occurs only in the iso-decay process of the 3rd stroke with the decay rate $\gamma_{\max}$. Then the shortest time for the system to achieve its steady state is $T_{\min} = 1/\gamma_{\max}$. If the evolution time of the 3rd stroke is $T = 12$ μs, the minimum decay rate for the chirality can be simply estimated as $\gamma_{\min}/4\Omega = (1/T)/4\Omega \approx 0.0276$. This complies with the numerical result in Fig.S. 5.

V. Nonreciprocal chirality

To further understand the relationship between the chirality and the topology, here we consider the encirclement restricted within single Riemann sheets. As such, we calculate the state evolution in clockwise and counterclockwise encirclements starting from $\Delta = 0$ and $\gamma = \gamma_{\min}$ as depicted in Figs.S. 6 and 7. We choose $t_d = 6$ μs and $t_y = 150$ μs to keep the change rate of the parameters consistent with our experimental conditions (Here t_d and t_y represent the durations of iso-decay and isochoric strokes, respectively).

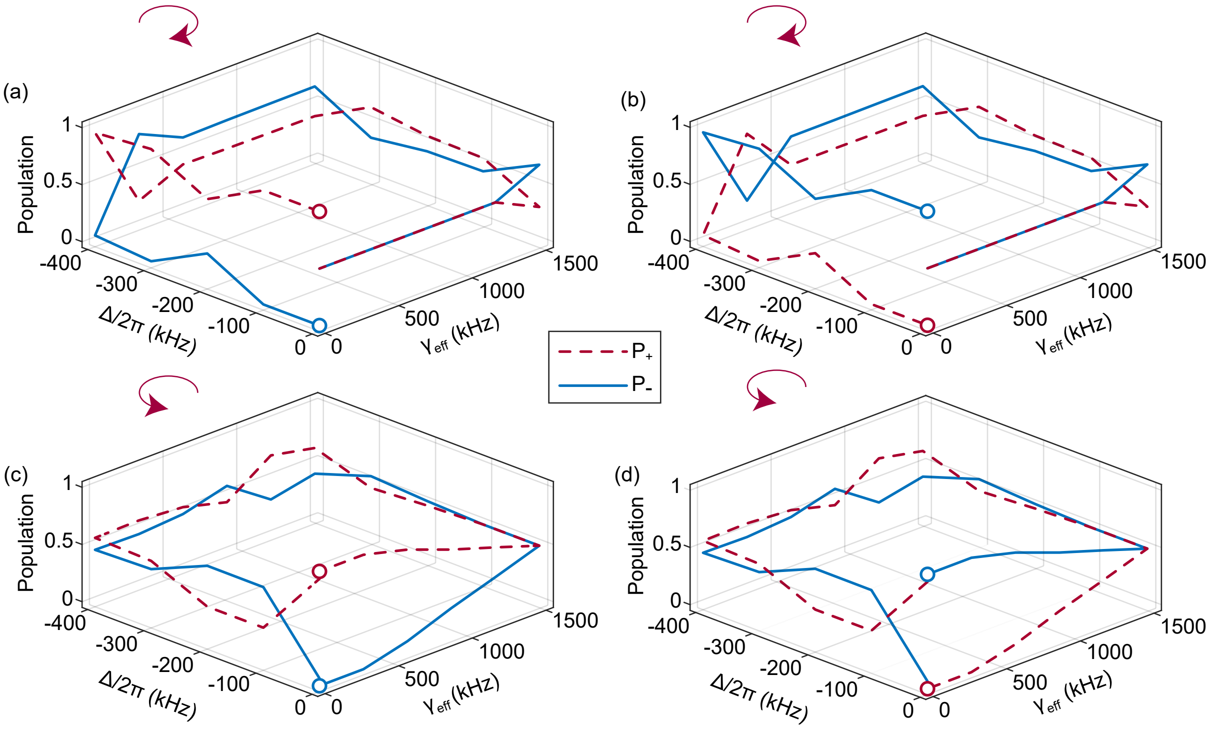

Fig.S. 6. Populations P_+ and P_- versus Δ and γ_{eff} in clockwise and counterclockwise encirclements, where the initial states $|\psi_+\rangle$ and $|\psi_-\rangle$ are considered respectively, and the typical parameters take the values of $\Delta_{\rm min}/2\pi = -400$ kHz, $\Delta_{\rm max}/2\pi = 0$ kHz, $\gamma_{\rm min} = 0$ kHz, and $\gamma_{\rm max} = 1.45$ MHz. The empty circles represent the starting points.

For the clockwise encirclements in Figs.S. 6(a, b), the initial state $|\psi_+\rangle$ or $|\psi_-\rangle$ evolves into a steady state that is very similar to the mixture state $(|e\rangle\langle e| + |g\rangle\langle g|)/2$ during the fourth stroke. So neither the closed encirclement nor asymmetric mode convention would appear in this case. In contrast, for the counterclockwise encirclements starting from either $|\psi_+\rangle$ or $|\psi_-\rangle$ as depicted by Figs.S 6(c, d), the system evolves to a steady state at the end of the third stroke, which is very close to the pure state $|g\rangle$ under the condition of large detuning. This steady state experiences a LZS with the detuning increasing from $\Delta/2\pi = -400$ kHz to $\Delta = 0$ kHz, reaching $|\psi_+\rangle$ finally. Therefore, in this case, starting from $|\psi_+\rangle$ yields a closed encirclement, and starting from $|\psi_-\rangle$ presents asymmetric mode convention.

For the clockwise encirclements in Figs.S 7(a, b), the initial state $|\psi_\pm\rangle$ evolves into a steady state at the end of the third stroke and finally reaches $|\psi_-\rangle$. So the initial state $|\psi_-\rangle$ leads to a closed encirclement, and starting from $|\psi_+\rangle$ presents asymmetric mode convention. In contrast, for the counterclockwise encirclements in Figs.S. 7(c, d), the system from either $|\psi_+\rangle$ or $|\psi_-\rangle$ finally evolves to a mixed state very similar to $(|e\rangle\langle e| + |g\rangle\langle g|)/2$. So both the closed encirclement and asymmetric mode convention would not appear in this case.

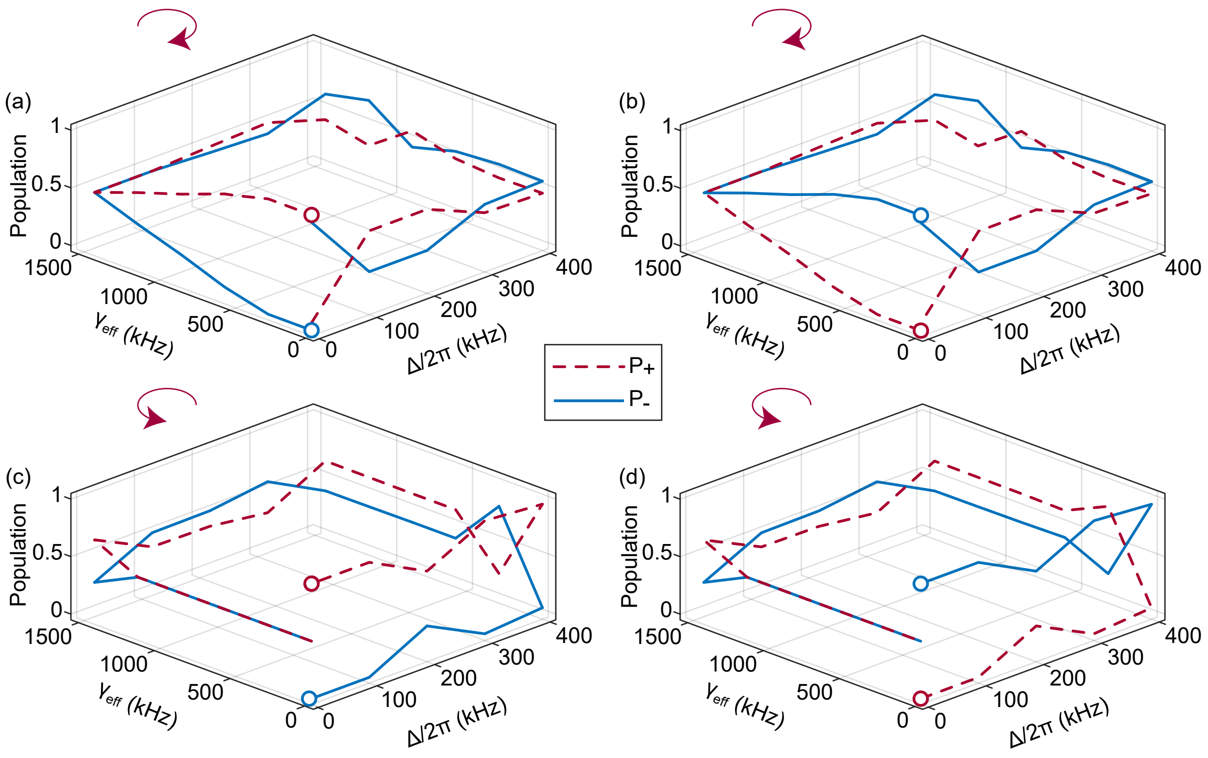

Fig.S. 7. Population P_+ and P_- versus Δ and γ_{eff} in clockwise and counterclockwise encirclements, where the initial states $|\psi_+\rangle$ and $|\psi_-\rangle$ are considered respectively, and the typical parameters take the values of $\Delta_{\rm min}/2\pi = 0$ kHz, $\Delta_{\rm max}/2\pi = 400$ kHz, $\gamma_{\rm min} = 0$ kHz, and $\gamma_{\rm max} = 1.45$ MHz. The empty circles represent the starting points.

Therefore, when the encirclements are accomplished within single Riemann sheets, the chirality is nonreciprocal and incomplete, which can be called non-reciprocal chirality, i.e., unidirectional chirality.

VI. More discussions about the chiral behavior and asymmetric mode conversion

Now, we numerically explore the impacts of the evolution time and dissipation on chiral behavior and asymmetric mode conversion. After a long-time evolution of the previous four strokes, our system decays to its steady state. Then, the evolution time of the fifth stroke will govern the following evolution and behavior as the Landau-Zener transitions. To clarify the above phenomena, we plot the fidelity $\langle\psi_\pm|\rho(T_5)|\psi_\pm\rangle$ versus the total evolution time of the fifth strokes T_5 as in Fig.S. 8.

In the absence of dissipations, the evolution time of the fifth stroke plays a crucial role in determining the adiabaticity. In the non-adiabatic regime, where the evolution time is very short, the system lacks enough time to follow the variation of the detuning. In this situation,

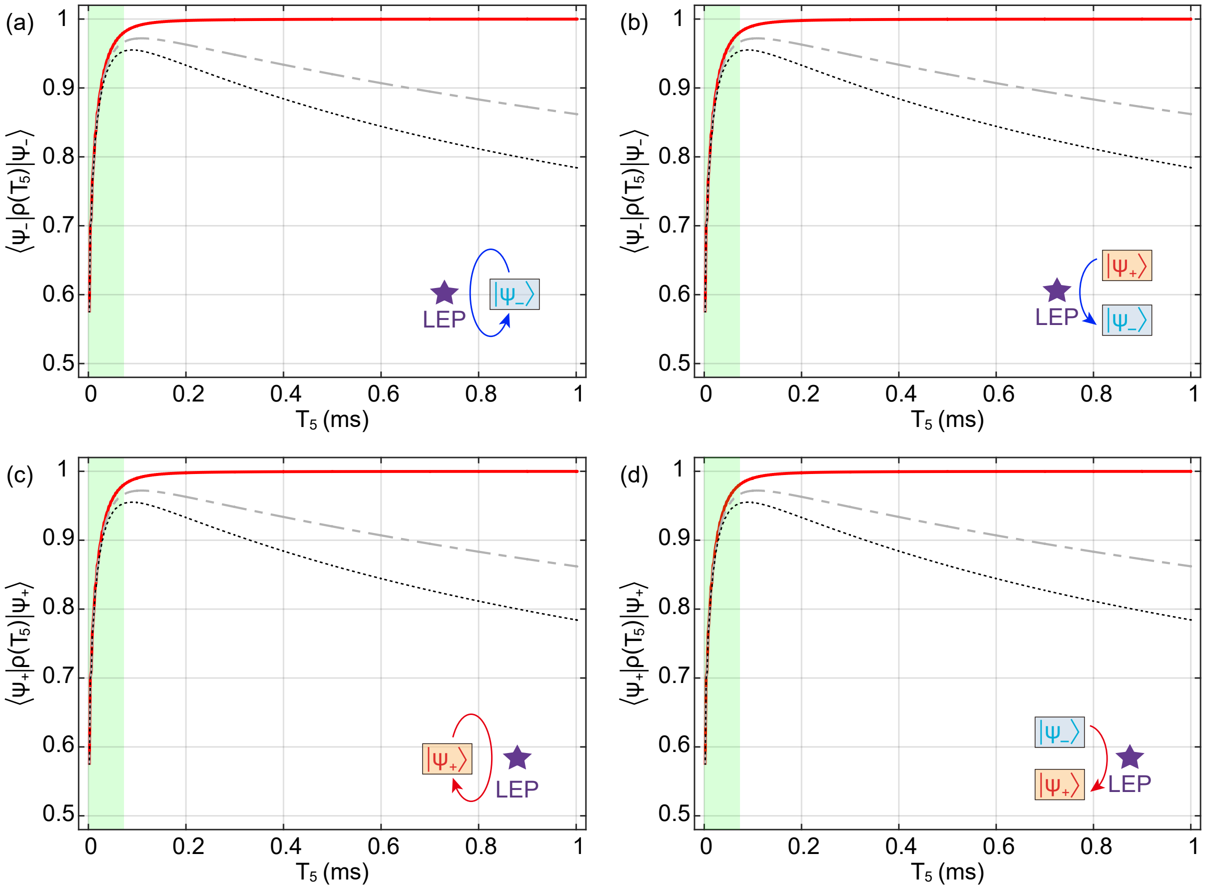

Fig.S. 8. Fidelity $\langle\psi_\pm|\rho(T_5)|\psi_\pm\rangle$ versus the total evolution time T_5 in the clockwise and counter-clockwise directions, with different initial states $|\psi_\pm\rangle$ and quantum jumps. We set $\gamma_{\min} = 0$ kHz for the red solid curve, $\gamma_{\min} = 25$ kHz for the grey dashed-dotted curve, and $\gamma_{\min} = 50$ kHz for the black dotted curve. Other parameters are $\Delta_{\min}/2\pi = -400$ kHz, $\Delta_{\max}/2\pi = 400$ kHz, $\gamma_{\min} = 0$ kHz, and $\gamma_{\max} = 1.45$ MHz.

non-adiabatic transitions between two eigenstates can occur, leading to variations in the accumulated phase. These phase variations can generate unexpected quantum interference of the Landau-Zener-Stückelberg process and weaken the effects of chiral behavior and asymmetric mode conversion (see the shadow areas in green in Fig.S. 8).

In contrast, in the adiabatic regime with a long enough evolution time, the system smoothly follows the detuning variation, and the accumulated phase approaches a fixed value. Therefore, adiabatic evolution can suppress non-adiabatic transitions and ensure the manifestation of chiral behavior and asymmetric mode conversion (see the red curves for $\gamma_{eff} = 0$ kHz in Fig.S. 8).

However, all experimental results are inevitably associated with dissipations, which lead to quantum jumps. The dissipations in the quantum system lead to a loss of coherence and introduce additional non-adiabatic processes to the ideal Landau-Zener transition. Conse-

quently, dissipations of the system affect the chiral behavior and asymmetric mode conversion. When we set a fixed evolution time, the increased dissipations adjust the chiral behavior towards non-chiral behavior by non-adiabatic transitions (see the gray dashed-dotted cures for $\gamma_{eff} = 25$ kHz and black dotted cures for $\gamma_{eff} = 50$ kHz in Fig.S. 8).

As a result, our experiments demonstrate chiral behavior and asymmetric mode conversion in general cases, which involve a fixed time and fixed dissipation in the iso-decay strokes. Additionally, the imperfect chiral behavior and asymmetric mode conversion arise from the fact that the conditions for two isochoric strokes cannot follow the conditions for the large detuning.

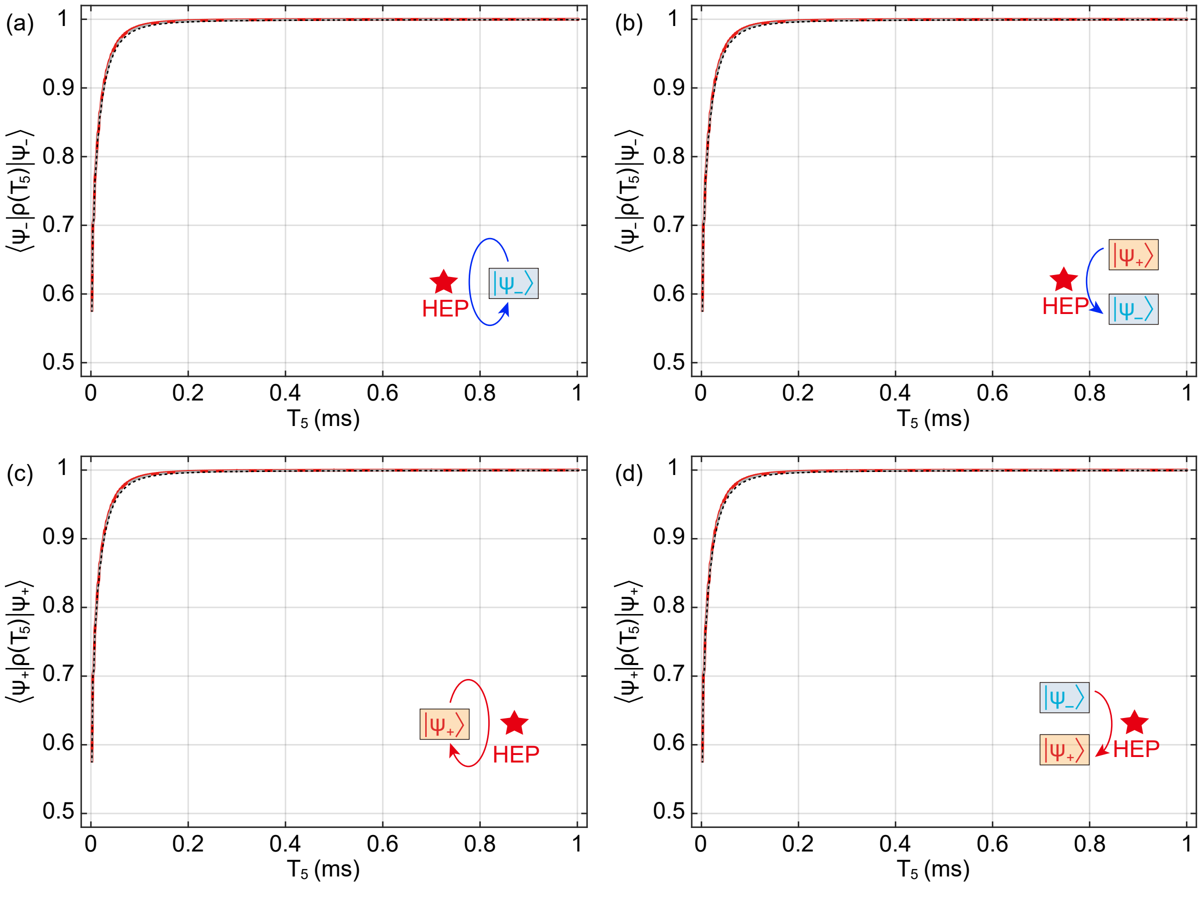

Fig.S. 9. Fidelity $\langle\psi_\pm|\rho(T_5)|\psi_\pm\rangle$ versus the total evolution time T_5 in the clockwise and counter-clockwise directions, with different initial states $|\psi_\pm\rangle$ and no quantum jumps. We set $\gamma_{\min} = 0$ kHz for the red solid curve, $\gamma_{\min} = 25$ kHz for the grey dashed-dotted curve, and $\gamma_{\min} = 50$ kHz for the black dotted curve. Other parameters are $\Delta_{\min}/2\pi = -400$ kHz, $\Delta_{\max}/2\pi = 400$ kHz, $\gamma_{\min} = 0$ kHz, and $\gamma_{\max} = 1.45$ MHz.

On the other hand, with quantum jumps ignored, the above results for LEPs return to those for HEPs. Then, we plot the corresponding results for HEP in Fig.S. 9. These figures demonstrate that, without the influence of quantum jumps, we can achieve perfect chiral

behavior and asymmetric mode conversion when the evolution time T_5 is long enough.

These phenomena illustrate that quantum jump is indeed a key quantum feature of the LEP, and our observed chiral behavior and asymmetric mode conversion result from the presence of the LEP.

---

[1] Wang, W. C. et al. Observation of PT -symmetric quantum coherence in a single-ion system. Phys. Rev. A 103, L020201 (2021).

[2] Chen, W., Abbasi, M., Joglekar, Y. N., & Murch, K. W., Quantum Jumps in the Non-Hermitian Dynamics of a Superconducting Qubit. Experimental Demonstration of Spontaneous Chirality in a Nonlinear Microresonator, *Phys. Rev. Lett.* **127**, 140504 (2021).

[3] Naghiloo M., Abbasi M., Joglekar Y. N., & Murch K. W., Quantum state tomography across the exceptional point in a single dissipative qubit, *Nat. Phys.* **15**, 1232 (2019).

[4] Chen, W., Abbasi, M., Joglekar, Y. N. & Murch, K. W. Quantum Jumps in the Non-Hermitian Dynamics of a Superconducting Qubit. Experimental Demonstration of Spontaneous Chirality in a Nonlinear Microresonator, *Phys. Rev. Lett.* **127**, 140504 (2021).

[5] Naghiloo M., Abbasi M., Joglekar Y. N., & Murch K. W., Quantum state tomography across the exceptional point in a single dissipative qubit, *Nat. Phys.* **15**, 1232 (2019).

[6] In this model, we can engineer both the Rabi frequency Ω and the effective dacay rate $\gamma_{\text{eff}} = \tilde{\Omega}^2/\Gamma$ under the condition of $\Omega \ll \tilde{\Omega}$ [7, 8]. With this level of controllability, we can fully tune this two-level system and perform parametric loops that encircle or do not encircle the LEP.

[7] Zhang J. W., Rehan K., Li M., Li J. C., Chen L., Su S. L., Yan L. L., Zhou F., & Feng M., Single-atom verification of the information-theoretical bound of irreversibility at the quantum level, *Phys. Rev. Research* **2**, 033082 (2020).

[8] Zhang J.-W., Zhang J.-Q., Ding G.-Y., Li J.-C., Bu J.-T., Wang B., Yan L.-L., Su S.-L., Chen L., Nori F., Özdemir Ş. K., Zhou F., Jing H., & Feng M., Dynamical Control of Quantum Heat Engines Using Exceptional Points, *Nat. Commun.* **13**, 6225 (2022).
